# App-Based Intervention Studies for Family Caregivers of Older Adults: A Scoping Review

**DOI:** 10.1093/geroni/igaf122.1139

**Published:** 2025-12-31

**Authors:** Xin Yao Lin, Francesca Falzarano, E-Shien Chang, Kristin Hon, Ruoying Zhang, Andy Hickner, Sara Czaja

**Affiliations:** Weill Cornell Medicine, New York, New York, United States; University of Southern California, Los Angeles, California, United States; Weill Cornell Medicine, New York, New York, United States; Weill Cornell Medicine, New York, New York, United States; Boston College, Chestnut Hill, Massachusetts, United States; Weill Cornell Medicine, New York, New York, United States; Weill Cornell Medicine, New York, New York, United States

## Abstract

As the global population ages, the increasing number of individuals with chronic conditions places a growing burden on family caregivers. Behavioral interventions delivered via app-based interventions, including apps on mobile phones, tablet, or web, have emerged as a powerful tool for enhancing caregiver support. The current study aims to identify and describe app-based interventions for family caregivers of older adults with chronic conditions, focusing on their designs, features, and impact on caregiver outcomes. This review is reported according to the Preferred Reporting Items for Systematic Reviews and Meta-analyses (PRISMA). A search of publications from 2007 through December 20, 2022, was conducted across PsycINFO (EBSCO), MEDLINE ALL (Ovid), Embase (Ovid), Web of Science Core Collection (A&HCI, BKCI-SSH, BKCI-S, CCR-EXPANDED, ESCI, IC, CPCI-SSH, CPCI-S, SCI-EXPANDED, SSCI) (Clarivate), ACM Guide to Computing Literature (ACM Digital Library), and Engineering Village (Elsevier), using relevant keywords. The database search identified 9,482 studies, resulting in 290 full texts and a final 49 studies included. Forty-four unique apps were identified, demonstrating different designs and features. These studies primarily examined the impact of apps on caregiver well-being, burden, and mental health. While the majority of interventions were perceived as beneficial, several design and research limitations were noted. While many interventions demonstrate positive effects on caregiver outcomes, there are significant gaps in research design, evaluation, and reporting. Future studies should prioritize more integration of self-care practices, clarity in intervention components, rigorous adherence to behavioral intervention frameworks, and greater inclusivity in participant samples.

